# When increasing distraction helps learning: Distractor number and content interact in their effects on memory

**DOI:** 10.3758/s13414-017-1399-1

**Published:** 2017-08-10

**Authors:** Kate Nussenbaum, Dima Amso, Julie Markant

**Affiliations:** 1 Department of Experimental Psychology, University of Oxford, Parks Road, Oxford, UK OX1 3PH; 2 Department of Cognitive, Linguistic, and Psychological Sciences, Brown University, Providence, RI USA; 3 Department of Psychology, Tulane University, New Orleans, LA USA

**Keywords:** Attention: Selective, Attention: Interactions with memory, Memory: Longterm memory

## Abstract

Previous work has demonstrated that increasing the number of distractors in a search array can reduce interference from distractor content during target processing. However, it is unclear how this reduced interference influences learning of target information. Here, we investigated how varying the amount and content of distraction present in a learning environment affects visual search and subsequent memory for target items. In two experiments, we demonstrate that the number and content of competing distractors interact in their influence on target selection and memory. Specifically, while increasing the number of distractors present in a search array made target detection more effortful, it did not impair learning and memory for target content. Instead, when the distractors contained category information that conflicted with the target, increasing the number of distractors from one to three actually *benefitted* learning and memory. These data suggest that increasing numbers of distractors may reduce interference from conflicting conceptual information during encoding.

## Introduction

As we navigate our complex visual world, different stimuli compete for our limited processing capacity. Selective attention enables us to prioritize processing in favor of relevant items while suppressing irrelevant distractors (Carrasco, 2011; Desimone & Duncan, 1995; Gazzaley, Cooney, McEvoy, Knight, & D’Esposito, 2005; Kastner & Ungerleider, 2001; Moran & Desimone, 1985; Reynolds, Chelazzi, & Desimone, 1999). We need to suppress distraction not only to pay attention and adaptively respond to relevant stimuli in our environments (Gaspar & McDonald, 2014; Geng, 2014), but also to encode the details of the world around us that are most likely to be useful later (Astle, Nobre, & Scerif, 2012; Markant, Worden, & Amso, 2015; Toepper et al., 2010).

Understanding the relationship between visual distraction and memory is critical in order to design effective learning environments and intervention strategies to enhance learning and memory in individuals who may struggle to encode relevant information. Though our intuition may suggest that the best learning environments are those with the fewest number of distractors, it is unclear how the nature of the visual distractors around us causes them to interfere with or facilitate our ability to selectively learn from our environments.

Here we examine how varying the amount and the content of distractors influences individuals’ ability to selectively encode relevant information, with the hypothesis that the attentional allocation to distractors at encoding will affect the degree to which target items are learned and remembered.

### Distractor suppression underlies successful memory encoding

Previous research has shown that individuals are better at remembering attended versus ignored information (Gazzaley et al., 2005). This relationship holds true for both explicit and implicit memory and across different delay periods (Ballesteros, Reales, García, & Carrasco, 2006). Critically, the extent to which people enhance their attention toward selected information is not the only attention-related predictor of future memory success. Effective memory encoding also relies on *suppression* of irrelevant information. The degree to which individuals are able ignore distraction predicts their ability to hold selected information in mind, as indexed by short-term and working memory tasks (Astle, Nobre, & Scerif, 2012; Gulbinaite, Johnson, de Jong, Morey, & van Rijn, 2014; Zanto & Gazzaley, 2009). Additionally, the beneficial effects of distractor suppression on memory encoding extend into the realm of long-term memory, with individuals demonstrating enhanced memory for target items that were encoded while a competing distractor location was simultaneously suppressed relative to target items that were encoded in the absence of concurrent suppression (Markant et al., 2015).

### Multiple distractors may be *less* distracting

Previous work has demonstrated that distractor suppression promotes successful short- and long-term memory encoding, but it is unclear how the presence of multiple distractors in a visual environment shifts attention dynamics and influences recognition memory for target information.

Two related bodies of work have explored the effects of distractor number on visual attention. First, visual search studies have found that efficient visual search involves the active suppression of neural activity related both to irrelevant distractor categories (Seidl, Peelen, & Kastner, 2012) and to irrelevant distractor locations (Wang & Klein, 2010). Additionally, individuals can simultaneously suppress multiple competing distractor locations (Dodd, Van der Stigchel, & Hollingworth, 2009; Mazza, Turatto, & Caramazza, 2009; Wang & Klein, 2010), with some studies suggesting that people are able to suppress stimuli presented in up to five previously encountered locations (Dodd et al., 2009; Snyder & Kingstone, 2000; Tipper, Weaver, & Watson, 1996). However, the effects of suppressing one versus multiple distractor locations on target encoding and recognition memory have, to the best of our knowledge, never been examined.

Second, studies of perceptual load have found that more cluttered search arrays can reduce interference from the specific content of individual distractors (Forster & Lavie, 2008; Lavie, Hirst, de Fockert, & Viding, 2004; Lavie & Tsal, 1994; Torralbo & Beck, 2008). In these studies, participants typically locate and categorize a target stimulus hidden among distractors. For example, in Lavie and Cox (1997), participants searched for an “X” or an “N” within a circular search array of letters. A compatible (i.e., an “N” when the search target was an “N”), incompatible (“X” when the search target was an “N”), or neutral distractor (“Y” when the search target was either an “X” or an “N”) was presented adjacent to the search array. Participants saw search arrays with zero, one, three, and five distractors. As the number of distractors included in the array increased, participants were slower to identify the target across all compatibility trial types. When the array contained only a single distractor, participants also demonstrated a distractor compatibility effect; that is, their target categorization was slowed by incompatible relative to neutral distractors presented adjacent to the search array. However, this compatibility effect disappeared when there were five distractors present in the search array, indicating that participants experienced less interference from the specific content of the distractors even though the overall visual search was more difficult. In other words, when it was easier for participants to locate the target within the array, the specific content of the adjacent distractor had a greater effect on their performance.

The visual search and perceptual load literature thus suggest that individuals are capable of simultaneously suppressing multiple distractors and that doing so may actually reduce interference from any single distractor during visual processing of a target stimulus. Taken together with previous work on the role of distractor suppression during memory encoding, such findings suggest that increasing distraction may result in *better* memory for targets encoded in the context of an increasing number of distractors.

To test this question, we examined whether increasing distraction would reduce interference from any single distractor during visual search and ultimately enhance learning and memory for target images. In Experiment 1, participants performed a search task where they identified a target from among zero, one, or three distractor images and categorized target images as either “alive” or “not alive.” We predicted that increasing the number of distractors would make visual search more difficult and engage more effortful selective attention, as indexed by longer reaction times both to visually locate and to categorize the targets on the screen. We additionally predicted that this increased engagement of selective attention would lead to stronger subsequent memory for target images incidentally encoded in the context of greater numbers of distractors. In other words, we expected that the presence of more distraction would *benefit* learning and memory for the target image.

## Experiment 1

### Methods

#### Participants

Sixty-two adults (*M*_*Age*_ = 20.41 years, *SD*_*Age*_ = 1.95 years, 22 M) participated in the study. Participants were recruited from the community via advertisements and from Brown University’s undergraduate subject pool. According to self-report, 10% of participants identified as African American, 18% identified as Asian, 60% identified as Caucasian, 7% identified as Hispanic, and 11% identified as other or two or more races. Participants were compensated with either money or course credit. All participants gave informed consent prior to participation.

#### Experimental stimuli and procedures

**Fig. 1:**
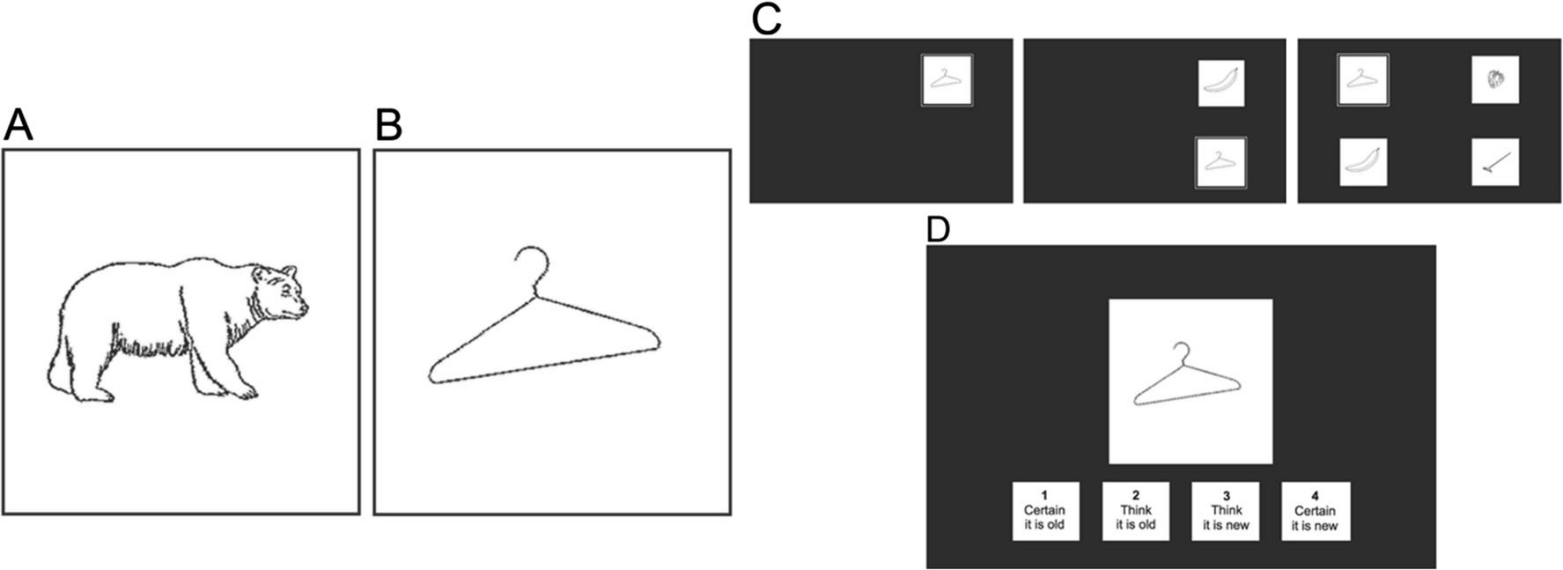
Examples of (**a**) alive and (**b**) not alive target and distractor images presented in the incidental encoding task. (**c**) Schematic example of encoding trial in the baseline, one-, and three-distractor conditions. The distractors flashed at a rate of 350 ms. (**d**) Example of recognition memory test trial

Stimuli for the experimental task included black line drawings of everyday objects against a white background, taken from a database of standardized images developed by Snodgrass and Vanderwart (1980). In total, 200 pictures were used. Sixty of these images served as target stimuli, 80 served as distractor stimuli, and 60 served as novel images during the recognition memory test. Half of the images in each group of stimuli could be categorized as “alive” (e.g., bear, alligator; Fig. 1a) and half could be categorized as “not alive” (e.g., hanger, saltshaker; Fig. 1b).

On every trial, participants first saw a 1,000-ms white fixation cross in the center of a 22-in. black screen. A 200 × 200 pixel (4.6° × 4.6°) target image outlined in a thin, white box then appeared in one of the screen’s four quadrants. Depending on the distractor condition (baseline, one distractor, three distractor), zero, one, or three peripheral distractor images appeared across the remaining three quadrants (Fig. 1c). Target images remained on the screen for 2,100 ms in all conditions. In the one-distractor and three-distractor conditions, the peripheral distractor images appeared concurrently with the target image, remained on the screen for 350 ms, then disappeared for 350 ms. Within each trial, the same distractor images and continued to flash on and off at this rate for the full 2,100 ms. The three-distractor condition was identical to the one-distractor condition except that three different peripheral distractor images flashed in synchrony in all three of the non-target quadrants. Different distractors were presented on every trial. The experimental timings were chosen after behavioral piloting to ensure that participants would have enough time to visually locate and respond to the target and demonstrate above-chance memory performance.

Participants were instructed to indicate whether the target image (outlined in white) was “alive” or “not alive” by pressing “1” or “2” as quickly and as accurately as possible. Participants completed 20 trials in each condition, for a total of 60 trials.^1^ Participants were not instructed to study or memorize the target or distractor images. Trial order was randomized for each participant, with the three different distractor conditions interleaved rather than blocked. The orders of the target and distractor image presentation within each condition were also randomized. Target images were counterbalanced across participants so that they appeared in each condition an equal number of times (i.e., as a target in the baseline, one distractor, and three-distractor conditions or as a distractor in the one and three-distractor conditions). The target and distractor locations were also counterbalanced across condition for each participant.

Following the search/incidental encoding task, participants completed an unexpected test of recognition memory. Participants saw all of the target and distractor images they had previously seen as well as 60 novel images and were asked to determine on a scale of 1 to 4 whether they were (1) certain the image was old, (2) thought the image was old, (3) thought the image was new, or (4) were certain the image was new (Fig. 1d). On every test trial, a 200 × 200-pixel (4.6° × 4.6°) image appeared in the center of the screen, with the four possible responses written underneath to remind participants of which keys corresponded to which memory decisions. The images appeared in a random order at test. Participants had 5 s to make each response.

#### Eye-tracking procedure

Eye movements were recorded using a remote eye tracker (SensoMotoric Instruments RED system). Participants sat 70 cm from the 22-in. monitor. At the beginning of the test session, each participant’s point of gaze (POG) was calibrated using a 5-point protocol provided by the SMI Experiment Center software. Four additional targets were presented following calibration to determine the accuracy of the POG calibration. This validation procedure provides a measure of deviation between each subject’s measured POG and the stimuli presented on the screen. For participants included in the eye-tracking analyses (N = 54, see below for exclusion criteria), average horizontal and vertical deviations were .99° (*SD* = .59°) and .99° (*SD* = .51°), respectively. After data collection was completed, four areas of interest (AOIs) were drawn using SMIs BeGaze Software. Each of these AOIs were 400 × 400 pixels (9.2° × 9.2°) and centered over the target and distractor image locations such that each AOI contained the entire area where images appeared as well as a 100-pixel border on each side of the image location. The horizontal visual angle separating the internal edges of the left and right AOIs was 10.2°. The vertical visual angle separating the internal edges of the top and bottom AOIs was 2.9°. Three participants were excluded from the eye-tracking analyses because their POG calibration deviations were greater than these values, and thus we could not be certain when they were looking at the quadrant that contained a target image or an adjacent image. An additional five participants did not contribute eye movement latency data on more than 50% of trials due to eye-tracker error; we also excluded data from these participants only from our eye-tracking analyses.

### Data processing

#### Visual search/encoding

We defined three variables that reflected the difficulty of target selection across conditions: eye-movement reaction time to the target, target verification time, and target categorization accuracy. Eye-movement reaction times were based on the time of the participant’s first fixation into the target image AOI following its onset. Looks were considered fixations if a participant’s POG remained within a 100-pixel area for at least 100 ms. Trials were excluded if a participant’s eye-movement reaction time was faster than 100 ms as such rapid eye movements were likely anticipatory rather than voluntary or reflexive movements in response to the stimulus onset (Klein, Foerster, Hartnegg, & Fischer, 2005). Target verification times were based on the time between the participant’s first fixation on the target on each trial and their manual response time on that trial, reflecting the speed with which they categorized the target after visually locating it. Only trials included in the eye-movement reaction time analysis were considered. Trials were further excluded from the target verification time analysis excluded if participants’ manual reaction times were faster than 200 ms, as these were likely to be anticipatory rather than planned motor responses (Woods, Wyma, Yund, Herron, & Reed, 2015). For both variables, to mitigate the effects of extreme, outlying values, we employed a standard trimming procedure in which trials were excluded if participants’ reaction times fell outside two standard deviations from their individual means. To calculate mean categorization accuracy, we computed the proportion of encoding trials in which participants successfully identified the category of the target image (alive or not alive) within each distractor condition.

We also defined three variables to characterize participants’ patterns of visual attention during encoding across distractor conditions: target looking time, total distractor looking time, and single distractor looking time. To compute each of these variables, we summed the amount of time participants looked at specific AOIs. For target looking time, we summed the amount of time participants looked at the target AOI on each trial. For total distractor looking time, we summed the amount of time participants looked at any of the other three non-target AOIs on each trial. These AOIs covered areas where distractors could appear. In order to fairly compare distractor looking times across distractor conditions, we examined fixations that fell within any of the three potential distractor AOIs on all trials, even in the baseline and one-distractor condition in which either none or only one of these AOIs was centered over a visual stimulus. We also examined whether participants focused on one distractor location or whether they distributed their attention over the three potential distractor locations during encoding. We computed single distractor looking time by determining the distractor location that each participant spent the most time looking at during each trial and summed their look durations within this AOI.

#### Recognition memory

We assessed recognition memory for target images using the confidence ratings participants provided at test. From these confidence ratings, we computed two measures of memory. First, we calculated memory sensitivity, *d*_*a*_, which was computed by fitting a receiver operator characteristic curve to the raw confidence data for each participant using the RSCORE PLUS algorithm (Harvey, 2013). These curves are based on signal detection theory, which assumes that an individual’s memory strength for an item lies somewhere along a continuous distribution that can be separated into “old” and “new” responses (e.g. Weidemann & Kahana, 2016). They are constructed by plotting the number of responses in each cumulative confidence bin (i.e., number of “certain it is old” responses, number of “certain it is old” + “think it is old” responses, number of “certain it is old” + “think it is old” + think it is new” responses, etc.) for old items versus new items. Memory sensitivity (*d*_*a*_), which reflects the distance between the old item hit rate and the new item false alarm rate, or in other words, the strength of the internal memory signal relative to noise, can be extracted from these curves. Second, we calculated the proportion of high confidence memory trials, which was the proportion of old images in each distractor condition which participants correctly identified as old with the highest confidence rating ("certain it is old"). We computed this latter measure based on previous work suggesting that examining high confidence trials only may be a more sensitive way to distinguish remembered from not-remembered images (Fukuda & Woodman, 2015; Krebs, Boehler, De Belder, & Egner, 2013) For both measures, test trials were excluded if participants failed to provide a response within 5 s, or if they pressed a key on the keypad that did not correspond to one of the four response options. On average, we excluded fewer than one memory test trial per participant (*M* = .97 trials, *SD* = 1.31 trials).

### Results

Unless otherwise stated, our analyses used repeated-measures ANOVA with distractor condition (baseline, one distractor, three distractor) as a within-subjects factor and the Greenhouse-Geisser correction as necessary to correct for violations of the assumption of sphericity. Significant interactions were followed with planned paired t-tests comparing participants’ attention and memory performance in the baseline versus one-, baseline versus three-, and one versus three-distractor conditions.

### Visual search

#### Eye-movement reaction times

**Fig. 2:**
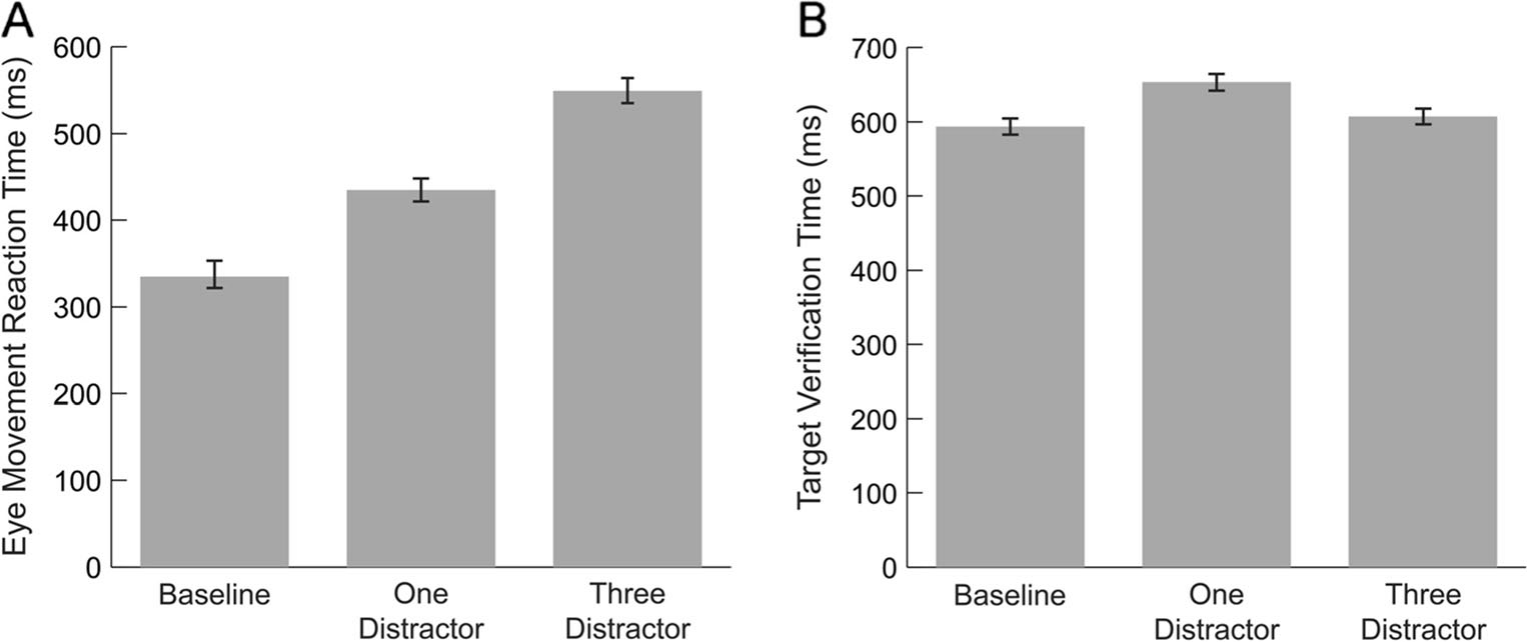
(**a**) Eye-movement reaction times to target images and (**b**) target verification times across distractor conditions. Error bars indicate standard error

We first examined the effects of increasing distractor number on target selection time during visual search by examining eye-movement reaction times to the target. Results of this analysis revealed a significant main effect of distractor condition, *F*(2, 106) = 161.26, *p* <.001, η^2^﻿ = .753 (Fig. 2), with reaction times showing a linear increase as the number of distractors on the screen increased (*M*_*0*_ = 335 ms, *SD*_0_ = 109 ms; *M*_*1*_ = 435 ms, *SD*_*1*_ = 91 ms; *M*_*3*_ = 550 ms, *SD*_*3*_ = 127 ms). Differences between all pairs of conditions were significant (all *p*s <.001).

#### Target verification time

We next examined how long it took participants to make a manual response after their first fixation on the target. Distractor condition affected target verification times, *F*(2, 106) = 10.63, *p* < .001, η^2^﻿ = .167 (Fig. 2b). However, unlike target latencies, verification times did not increase linearly with the number of distractors. Instead, participants were slowed by the presence of a single distractor (*M* = 653 ms, *SD* = 171 ms) relative to both the baseline condition (*M* = 594 ms, *SD* = 170 ms); *t*(53) = 4.54, *p* < .001, and the three-distractor condition (*M* = 607 ms, *SD* = 165 ms); *t*(53) = 3.22, *p* = .002. There was no difference in verification times between the baseline and three-distractor conditions, *t*(53) = 1.03, *p* = .306. Despite being faster to visually locate the target in the one relative to the three-distractor condition, participants were slower to categorize it after finding it, suggesting they may have experienced more interference from the single distractor as they processed the target.

#### Duration of looking

We also examined how visual attention was distributed across target and distractor locations for each condition. There was a significant effect of distractor condition on mean target looking times, *F*(1.76, 93.34) = 25.36, *p* < .001, η^2^﻿ = .324, with participants spending less time looking at the target as the number of distractors on the screen increased (*M*_*0*_ = 1,306 ms, *SD*_*0*_ = 409 ms; *M*_*1*_ = 1,157 ms, *SD*_*1*_ = 324 ms; *M*_*3*_ = 1,103 ms, *SD*_*3*_ = 331 ms). Target looking times were significantly different between all pairs of conditions (all *p*s < .027).

There was also a significant effect of distractor condition on the total amount of time that participants spent looking at distractor locations, *F*(2, 106) = 99.60, *p* < .001, η^2^﻿ = .653. Pairwise comparisons revealed that these differences were driven by participants spending less time looking at distractor locations in the baseline condition (*M* = 8 ms, *SD* = 23 ms) relative to the one-distractor condition (*M* = 192 ms, *SD* = 109 ms); *t*(53) = 12.16, *p* < .001, and the baseline condition relative to the three-distractor condition (*M* = 177 ms, *SD* = 117 ms); *t*(53) = 10.6, *p* < .001. We found no differences in total distractor looking between the one- and three-distractor conditions, *t*(53) = 1.25, *p* = .217. This indicates that participants did not spend significantly different amounts of time fixating on non-target locations across the one- and three-distractor conditions.

Distractor condition also affected single-distractor looking times, *F*(1.74, 92.09) = 111.98, *p <* .001, η^2^ = .679. Here however, participants spent more time fixating on any single distractor location in the one-distractor condition (*M* = 191 ms, *SD* = 109 ms) relative to both the baseline condition (*M* = 8 ms, *SD* = 21 ms) *and* the three-distractor condition (*M* = 150 ms, *SD* = 85; *p*s < .002). These results suggest that participants distributed their attention across multiple distractor locations during the three-distractor condition whereas they primarily attended only to the single distractor that was present on the screen during the one-distractor condition.

#### Categorization accuracy

Finally, there was no effect of distractor condition on target categorization accuracy, *F*(1.82, 110.778) = 2.51, *p* = .091, η^2^﻿ = .039, with participants performing well (>86% accuracy) across all three-distractor conditions.

### Recognition memory

#### Target images

**Fig. 3:**
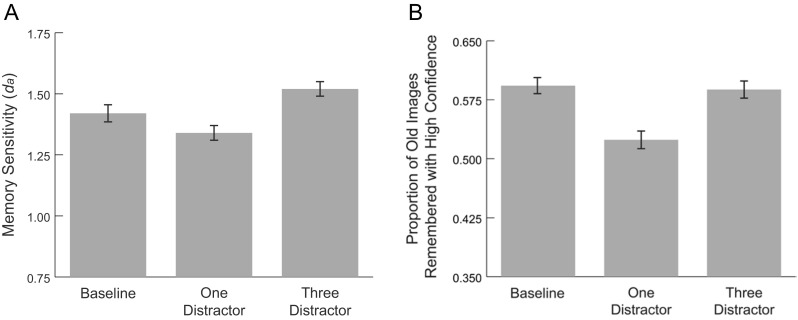
(**a**) Memory sensitivity (d_a_) and (**b**) the proportion of old images remembered with high confidence across distractor conditions. Error bars indicate standard error

The distractor manipulation during encoding had a significant effect on participants’ recognition memory sensitivity (*d*_*a*_) for target images at test, *F*(2, 122) = 3.61, *p* = .03, η^2^﻿ = .056 (Fig. 3a). Follow-up paired comparisons revealed that participants demonstrated significantly impaired memory sensitivity in the one-distractor condition (*M* = 1.34, *SD* = .50) relative to the three distractor-condition (*M* = 1.52, *SD* = .46); *t*(61) = 2.90, *p* = .006. Memory sensitivity in the baseline condition was not significantly different from either distractor condition (*p*s > .18).

Additionally, we observed an effect of distractor condition on the proportion of images remembered with high confidence, *F*(2, 122) = 6.42, *p* = .002, η^2^﻿ = .095 (Fig. 3b). Participants remembered significantly fewer images with high confidence in the one-distractor condition (*M* = .52, *SD* = .17) relative to the baseline condition (*M* = .59, *SD* = .16); *t*(61) = 3.33, *p* = .001, and relative to the three-distractor condition (*M* = .59, *SD* = .17), *t*(61) = 3.15, *p* = .003. There was no difference in the proportion of old images remembered with high confidence between the baseline and three-distractor conditions, *t*(61) = .25, *p* = .804. Thus participants demonstrated the weakest memory in the one-distractor condition, even though they were faster to visually locate the target *and* they spent more time looking at the target in this condition relative to the three-distractor condition. Despite the more difficult search process, participants were better able to encode the targets in the three- relative to the one-distractor condition.

#### Distractor images

Participants’ memory sensitivity (*d*_*a*_) for the distractors presented in the one-distractor condition (*M* = .35, *SD* = .38) was better than their memory sensitivity for the distractors presented in the three-distractor condition (*M* = .07, *SD* = .32), *F*(1, 61) = 49.6, *p* < .001, η^2^﻿ = .449. Additionally, one-sample t-tests revealed that distractor memory sensitivity in the one-distractor condition was above chance (0), *t*(61) = 7.2, *p* < .001, while distractor memory sensitivity in the three-distractor condition was not, *t*(61) = 1.8, *p* = .084. Participants also remembered a greater proportion of distractors in the one-distractor condition with high confidence, *F*(1, 61) = 53.4, *p* < .001, η^2^ = .467; *M*_*1*_ = .16, *SD*_*1*_ = .01; *M*_*3*_ = .07, *SD*_*3*_ = .01. These data indicate that participants were able to process and encode the single distractors to a greater degree than those that were present in the three-distractor condition.

### Experiment 1 discussion

The results of Experiment 1 showed that participants were slower to visually locate targets in the three- versus one-distractor condition, but that they were worse at encoding targets presented in the one relative to the three-distractor condition. This pattern of results suggests that the three-distractor condition predictably elicited greater attentional demands during search but that the single distractor uniquely interfered with participants’ ability to encode a target image. Furthermore, participants spent more time looking at a single distractor and were better able to remember the distractor images in the one- relative to the three-distractor condition, suggesting that they processed each distractor image more in the one- relative to the three-distractor condition. Participants’ slower verification time in the one- relative to the three-distractor condition similarly suggests that they may have experienced more interference from the single distractor. In contrast, in the three-distractor condition, participants’ divided attention across multiple distractors may have prevented them from processing any single distractor to the same extent that they processed the lone distractor in the one-distractor condition. Together, these data suggest that increased interference from distraction may have compromised learning and memory processes in the one-distractor condition, when *fewer* distractors were present.

These data are consistent with our initial hypothesis that increasing distraction from a single to multiple distractors during visual search can benefit learning and memory. We predicted this result based on two bodies of previous work that have shown both that memory benefits from selective attention engagement involving distractor suppression (Astle et al., 2014; Markant et al., 2015; Zanto & Gazzaley, 2009) and that busier search arrays with higher perceptual loads lead to increased competition among distractor stimuli, which ultimately prevents participants from processing the content of any single distractor (Lavie, Hirst, de Fockert, & Viding, 2004).

Critically, this previous work suggests that we may have seen poorer target recognition memory in the one-distractor condition because the visual search task was easy enough in this condition that participants could find the target and still process the *meaningful content* contained within the distracting image. Competition from the conceptual information contained within the distractors may have impaired memory to a greater extent in the one-distractor condition relative to the three-distractor condition, where the distractors were processed to a lesser degree. To test directly this idea, we conducted a second experiment using distractors that conflicted with the target category (alive vs. not alive), matched the target category, or contained no meaningful information (scrambled images). We predicted that, as in Experiment 1, increasing the number of distractors would make visual search more difficult and engage more effortful selective attention, indexed by slower response times to locate the target. However, unlike Experiment 1, we also predicted that the easier visual search in the one-distractor condition would enable processing of the information contained within the distractor, leading to slower target verification times and impaired target categorization accuracy when the distractor category conflicted with the target category in the one but not three-distractor condition. Furthermore, we expected that target memory would again be impaired in the one-distractor condition, but only when the distractors contained meaningful content.

## Experiment 2

### Methods

#### Participants

Fifty-three adults (*M*_*Age*_ = 22.2 years, *SD*_*Age*_ = 2.7 years, 21 M) participated in the study. As in Experiment 1, participants were recruited from the community via advertisements and from Brown University’s undergraduate subject pool. According to self-report, 9% of participants identified as African American, 23% identified as Asian, 58% identified as Caucasian, 11% identified as Hispanic, 2% identified as Native American, and 8% identified as other or two or more races. Participants were compensated with either money or course credit. All participants gave informed consent prior to participation.

#### Experimental stimuli and procedure

**Fig. 4:**
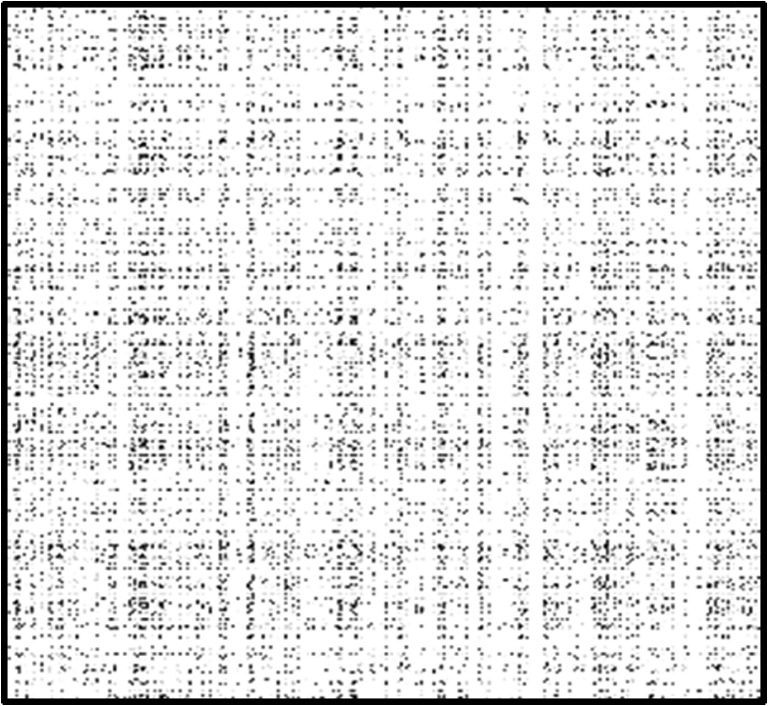
An example of a scrambled distractor image

In total, 40 scrambled images (Fig. 4) and 285 meaningful pictures served as experimental stimuli, from which 240 images per participant were used. Stimuli for the experimental task included some of the same black line drawings of everyday objects from Snodgrass and Vanderwart (1980) that were used in Experiment 1. In order to strengthen our distractor content analyses, images that may have confused subjects during the “alive” and “not alive” decision in Experiment 1 (e.g., plants) were not used in Experiment 2. In order to have sufficient images for Experiment 2, we added a selection of images from the International Picture Naming Database (Szekely et al., 2004). Scrambled image distractors were a random subset of 60 images from the larger stimulus set in which all of the pixels were randomly shuffled to distort the image content.

The primary difference between Experiment 1 and Experiment 2 was the nature of the distractor content; the trial procedure in the search/incidental encoding task and the recognition memory task were identical across both experiments. In Experiment 2, participants completed 90 search/incidental encoding trials – 30 in the baseline condition, 30 in the one-distractor condition, and 30 in the three-distractor condition. Within the one- and three-distractor conditions, participants experienced three different trial types: conflict, match, and scrambled distractor. During conflict trials, the categories of the target and the distractor(s) were different (e.g., the target was alive and the distractors were not). During match trials, the categories of the target and the distractor(s) were the same. During scrambled distractor trials, the scrambled images served as the distractors. Half of the images within each trial type in each distractor condition were categorized as “alive” and half were categorized as “not alive.” Trial order was randomized and target location was counterbalanced within each participant. The images that were used as targets and distractors in each of the conditions and novel images at test were counterbalanced across participants. The stimulus images drawn from the Snodgrass and Vanderwart (1980) set and the stimulus images drawn from the International Picture Naming Database were evenly distributed across the targets, distractors and novel images, and evenly distributed across distractor conditions and trial types within each distractor condition. The meaningful images appeared in a random order at test for a total of 240 test trials.

#### Eye-tracking procedure

The eye-tracking procedure was identical to that used in Experiment 1. We excluded two participants from our eye-tracking analyses because they were missing latency data on more than 50% of trials. For participants included in the eye-tracking analyses (N = 51), average horizontal and vertical deviations were .66° (*SD* = .27°) and .75° (*SD* = .24°), respectively.

#### Data processing

We computed eye-movement reaction times, target verification times, categorization accuracy, target looking time, total distractor looking time, single distractor looking time, recognition memory sensitivity (*d*_*a*_), and the proportion of old images remembered with high confidence as in Experiment 1. As in Experiment 1, we excluded memory test trials in which participants failed to respond within the 5-s time limit. On average, we excluded fewer than two test trials per participant (*M* = 1.87 trials, *SD* = 4.93 trials).

### Results

We first examined eye-movement reaction times and recognition memory sensitivity using repeated-measures ANOVAs with distractor condition (baseline, one-distractor, three-distractor) as our only within-subjects factor to determine if we replicated the basic effects of Experiment 1. As in Experiment 1, eye-movement reaction times increased linearly as the number of distractors on the screen increased, *F*(1.6, 81.1) = 147.77, *p* < .001, η^2^ = .747, *M*_*0*_ = 321 ms, *SD*_*0*_ = 100 ms; *M*_*1*_ = 431 ms, *SD*_*1*_ = 116 ms; *M*_*3*_ = 505 ms, *SD*_*3*_ = 109 ms. We also observed a significant effect of distractor condition on memory sensitivity (*d*_*a*_), *F*(2, 104) = 8.04, *p* = .001, η^2^﻿ = .134. Unlike in Experiment 1, this effect was driven by enhanced memory sensitivity in the baseline condition (*M* = 1.34, *SD* = .45) relative to both the one-distractor condition (*M* = 1.17, *SD* = .38); *t*(52) = 3.6, *p* = .001, and the three-distractor condition (*M* = 1.2, *SD* = .34); *t*(52) = 2.86, *p* = .006. Participants demonstrated no differences in memory sensitivity across the one- and three-distractor conditions, *t*(52) = .67, *p* = .507. However, we predicted *a priori* that memory performance would be moderated by the content of the distractor images.

As such, our subsequent analyses focused on the one and three-distractor conditions and included trial type as we were primarily interested in how distractor content and number would interact to impact participants’ visual search and memory performance. Unless otherwise stated, we used repeated-measures ANOVAs with Distractor Condition (one distractor, three distractor) and Trial Type (conflict, match, scrambled distractor) as within-subject factors.

### Visual search

#### Eye-movement reaction times

**Fig. 5:**
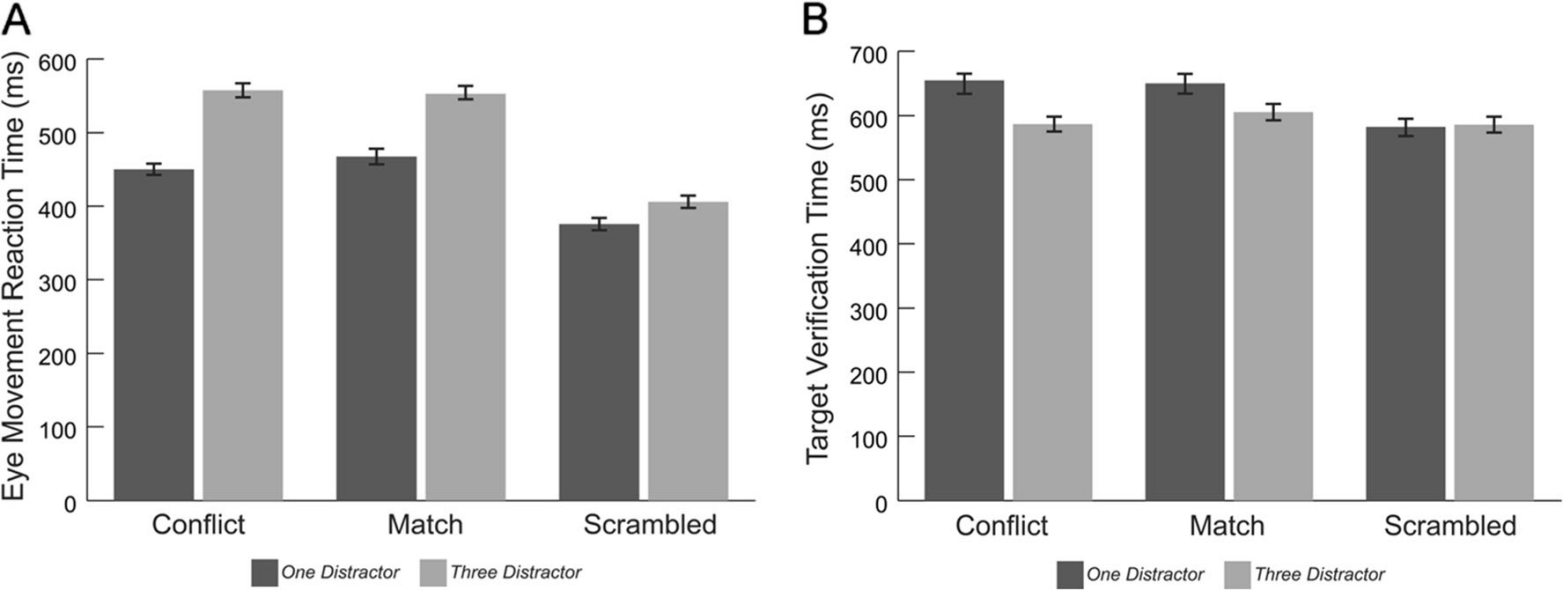
(**a**) Eye-movement reaction times and (**b**) target verification times across trial types and distractor conditions. Error bars indicate standard error

We next examined how distractor content and number influenced eye-movement reaction times. There was a main effect of distractor condition, *F*(1, 50) = 32.05, *p* <.001, η^2^﻿ = .391, reflecting slower reaction times in the three-distractor condition relative to the one-distractor condition (*M*_*1*_ = 431 ms, *SD*_*1*_ = 116 ms; *M*_*3*_ = 505 ms, *SD*_*3*_ = 109) (Fig. 5a). We also found a significant main effect of trial type, *F*(2, 100) = 85.04, *p* <.001, η^2^﻿ = .63, with slower reaction times for the conflict and match trials relative to the scrambled distractor trials (*M*_*C*_ = 504 ms, *SD*_*C*_ = 105 ms; *M*_*M*_ = 510 ms, *SD*_*M*_ = 123 ms; *M*_*S*_ = 391 ms, *SD*_*S*_ = 103 ms, all *p*s < .001). Finally, results also indicated a significant Distractor Condition × Trial Type interaction, *F*(1.8, 88.2) = 9.2, *p* < .001, η^2^﻿ = .155 (Fig. 5a). To probe this interaction, we computed the difference in reaction times between the three- and one-distractor conditions for each trial type. We then compared these differences. Participants were slowed to a greater degree by the increasing number of distractors during conflict trials (*M*_*Difference*_ = 107 ms, *SD* = 121 ms) and match trials (*M*_*Difference*_ = 85 ms, *SD* = 127 ms) relative to scrambled distractor trials (*M*_*Difference*_ = 30 ms, *SD* = 112 ms); *t*(50) = 3.68, *p* = .001, *t*(50) = 2.87, *p* = .006, respectively. There was no difference in the extent to which eye-movement reaction times were slowed by three distractors versus one distractor in conflict relative to match trials, *t*(50) = 1.49, *p* = .144.

#### Target verification times

We also examined how quickly participants categorized the target image after visually locating it. As in Experiment 1, there was a main effect of distractor condition, *F*(1, 50) = 7.20, *p* = .01, η^2^﻿ = .126 (Fig. 5b), reflecting slower verification times in the one-distractor condition relative to the three-distractor condition (*M*_*1*_ = 629 ms, *SD*_1_ = 197 ms; *M*_*3*_ = 593 ms, *SD*_3_ = 157 ms). Additionally, there was a main effect of trial type, *F*(2, 100) = 7.87, *p* = .001, η^2^﻿ = .136. Follow-up paired comparisons indicated that this effect was driven by faster verification times on scrambled distractor trials (*M* = 584 ms, *SD* = 166 ms) relative to both conflict trials (*M* = 621 ms, *SD* = 179 ms); *t*(50) = 3.00, *p* = .004, and match trials (*M* = 628 ms, *SD* = 188 ms); *t*(50) = 3.8, *p* < .001. We observed no differences in target verification times between conflict and match trials, *t*(50) = .59, *p* = .555.

These main effects were qualified by a significant distractor condition × trial type interaction, *F*(2, 100) = 3.77, *p* = .026, η^2^ = .07. To probe this interaction, we ran paired t-tests comparing verification times across each trial type within distractor conditions. In the three-distractor condition, verification times were equivalent across all trial types (*M*_*C*_ = 587 ms, *SD*_*C*_ = 164 ms; *M*_*M*_ = 606 ms, *SD*_*M*_ = 180 ms; *M*_*S*_ = 586 ms, *SD*_*S*_ = 175 ms), *p*s > .22. However, a different pattern emerged in the one-distractor condition. Here, participants demonstrated faster verification times in the scrambled distractor condition (*M* = 583 ms, *SD* = 190 ms) relative to both the conflict condition (*M* = 655 ms, *SD* = 222 ms); *t*(50) = 4.42, *p* <.001, and the match condition (*M* = 650 ms, *SD* = 218 ms), *t*(50) = 3.63, *p* = .001. Contrary to what we predicted, we observed no differences in verification times in the one-distractor condition between conflict and match trials, *t*(50) = .26, *p* = .798. Thus the main effect of trial type on target verification times were driven by participants being slowed by meaningful distractors, but only in the one-distractor condition.

#### Duration of looking

As in Experiment 1, participants spent more time looking at the targets in the one distractor relative to the three-distractor condition, *F*(1, 50) = 5.44, *p* = .024, η^2^﻿ = .096; (*M*_1_ = 1,347 ms, *SD*_1_ = 327 ms; *M*_3_ = 1,297 ms, *SD*_3_ = 314 ms). Additionally, there was a main effect of trial type, *F*(1, 100) = 25.85, *p* <.001, η^2^﻿ = .341, indicating that participants spent more time looking at the targets during scrambled distractor trials (*M* = 1,428 ms, *SD* = 366 ms), relative to both conflict trials (*M* = 1,287 ms, *SD* = 320 ms); *t*(50) = 5.35, *p* <.001, and match trials (*M* = 1252 ms, *SD* = 298 ms); *t*(50) = 6.10, *p* <.001. There was no difference in target looking times across conflict and match trials, *t*(50) = 1.57, *p* = .122, nor was there a distractor condition × trial type interaction, *F*(1, 100) = 1.00, *p* = .372, η^2^﻿ = .020.

Additionally, we examined the total time participants spent looking at any of the possible distractor locations across distractor conditions and trial types. As in Experiment 1, participants spent a statistically equivalent amount of time looking at distractor locations across the one- and three-distractor conditions, *F*(1, 50) = 0.00, *p* = .984, η^2^ = .00. Participants did however spend more time looking at distractor locations on conflict trials (*M* = 257 ms, *SD* = 158 ms) and match trials (*M* = 258 ms, *SD* = 138 ms) when the distractors contained meaningful content, relative to scrambled distractor trials (*M* = 109 ms, *SD* = 81 ms); *F*(1.77, 88.46) = 61.70, *p* < .001, η^2^﻿ = .552. There was no distractor condition × trial type interaction, *F*(2, 100) = .503, *p* = .606, η^2^﻿ = .010.

As in Experiment 1, a different pattern emerged when we examined single distractor looking times. The analysis revealed a main effect of distractor condition, *F*(1, 50) = 14.43, *p* < .001, η^2^﻿ = .224, with participants spending more time looking at any single distractor in the one-distractor (*M* = 207 ms, *SD* = 119 ms) versus three-distractor (*M* = 167 ms, *SD* = 85 ms) condition. There was also a main effect of trial type, *F*(1.76, 88.15) = 63.06, *p* < .001, η^2^﻿ = .558, with participants spending more time looking at any single distractor in the conflict and match conditions relative to the scrambled distractor condition, *p*s < .001; (*M*_*C*_ = 228 ms, *SD*_*C*_ = 131 ms; *M*_*M*_ = 227 ms, *SD*_*M*_ = 113 ms; *M*_*S*_ = 105 ms, *SD*_*s*_ = 77 ms)

#### Categorization accuracy

There was a main effect of distractor condition on categorization accuracy, *F*(1, 52) = 4.25, *p* = .044, η^2^ = .075, with participants demonstrating poorer accuracy in the one- relative to the three-distractor condition (*M*_*1*_ = .94, *SD*_*1*_ = .06; *M*_*3*_ = .95, *SD*_*3*_ = .05). Results also showed a main effect of trial type, *F*(2, 104) = 7.35, *p* = .001, η^2^ = .124, with participants performing more poorly during trials in which the distractors contained meaningful information relative to scrambled distractor trials, *p*s <.006; (*M*_*C*_ = .93, *SD*_*C*_ = .09; *M*_*M*_ = .94, *SD*_*M*_ = .05; *M*_*S*_ = .97, *SD*_*S*_ = .04). The Distractor Condition × Trial Type interaction was not significant, *F*(1.78, 95.89) = 2.730, *p* = .077, η^2^ = .050. These results suggest that participants experienced the most interference when a single distractor was present and when distractors contained meaningful information.

### Recognition memory

#### Target images

**Fig. 6:**
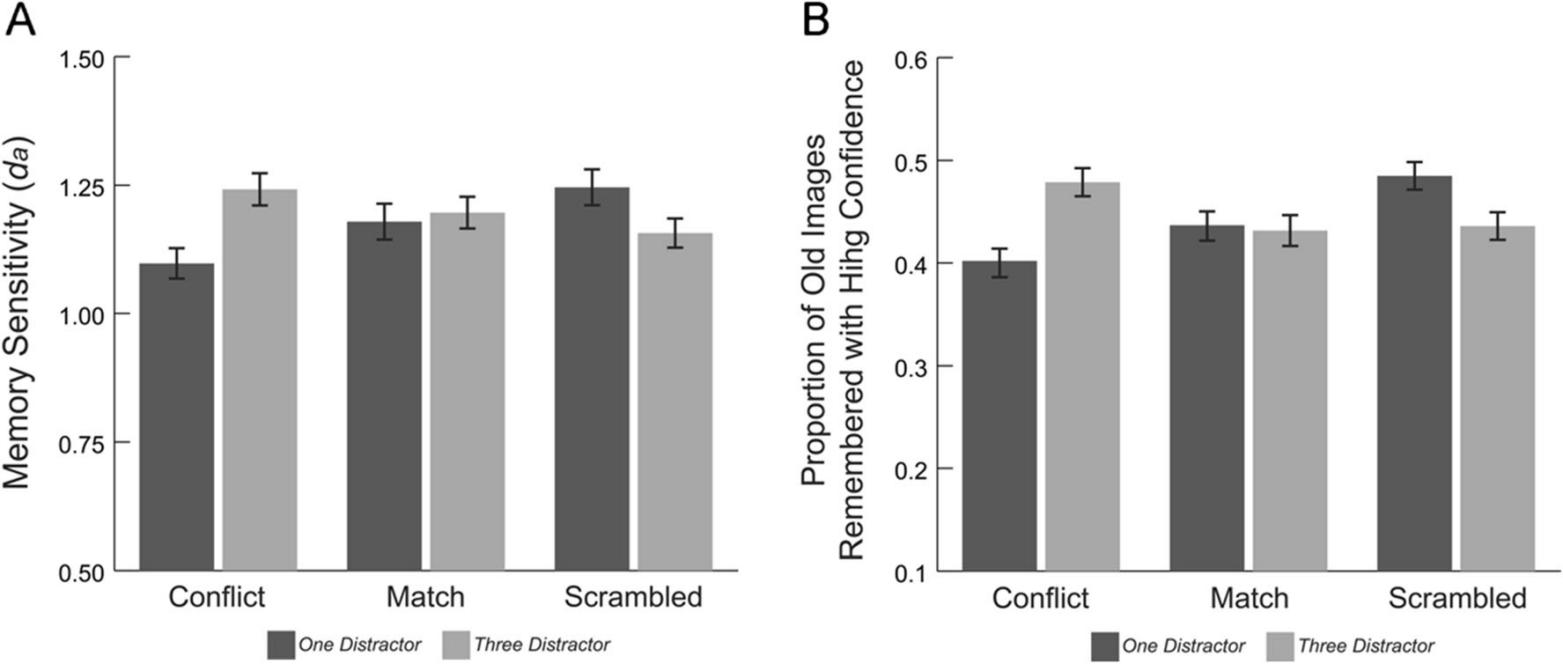
(**a**) Memory sensitivity (d_a_) and (**b**) the proportion of old images remembered with high confidence across distractor conditions and trial types. Error bars indicate standard error

Finally, we analyzed the effects of distractors on participants’ recognition memory. We first analyzed participants’ memory sensitivity (*d*_*a*_) across distractor conditions and trial types. No main effects or interactions reached significance (all *ps* > .067). However, given our a priori prediction that poorer memory performance would be specific to the meaningful trials during the one-distractor condition, we conducted planned comparisons examining memory sensitivity during conflict and match trials across the one- and three-distractor conditions. Results indicated that participants showed impaired memory in the one- relative to the three-distractor condition during conflict trials only (*M*_1C_ = 1.1, *SD*_1C_ = .44; *M*_3C_ = 1.24, *SD*_3C_ = .46); *t*(52) = 2.2, *p* = .032 (Fig. 6a). There was no difference in memory sensitivity for match trials, *t*(52) = .25, *p* = .803.

Additionally, we examined the effects of distractor condition and trial type on the proportion of old images that participants remembered with high confidence. Here, we observed a significant Distractor Condition × Trial Type interaction, *F*(2, 104) = 4.15, *p* = .018, η^2^﻿ = .074 (Fig. 6b). Follow-up paired t-tests revealed that participants remembered with high confidence a significantly greater proportion of the images presented during conflict trials in the three- relative to the one-distractor condition (*M*_1C_ = .40, *SD*_1C_ = .21; *M*_3C_ = .48, *SD*_3C_ = .20); *t*(52) = 2.81, *p* = .007. As with memory sensitivity, there was no statistical difference in the proportion of images participants remembered with high confidence on match trials across the distractor conditions (*M*_1M_ = .44, *SD*_1M_ = .22; *M*_3M_ = .43, *SD*_3M_ = .22); *t*(52) = .17, *p* = .862, or on scrambled distractor trials across the conditions (*M*_1S_ = .49, *SD*_1S_ = .20; *M*_3S_ = .44, *SD*_3S_ = .20); *t*(52) = 1.64, *p* = .107.

#### Distractor images

As in Experiment 1, participants showed greater memory sensitivity (*d*_*a*_) for the distractors in the one-distractor condition (*M* = .36, *SD* = .34) relative to the three-distractor condition (*M* = .13, *SD* = .24), *F*(1, 52) = 25.94, *p* <.001, η^2^﻿ = .333. There were no effects of trial type on distractor memory sensitivity, *F*(1, 52) = .63, *p* = .43, η^2^﻿ = .012, nor was there a distractor condition × trial type interaction, *F*(1, 52) = 0.00, *p* = .99, η^2^﻿ = 0.00. The same pattern held when we examined the proportion of distractor images remembered with high confidence. Participants remembered with high confidence a greater proportion of the distractor images presented in the one-distractor condition (*M* = .14, *SD* = .12) relative to the three-distractor condition (*M* = .07, *SD* = .05), *F*(1, 52) = 32.47, *p* < .001, η^2^﻿ = .384. There were no effects of trial type on the proportion of images remembered with high confidence, *F*(1, 52) = 3.03, *p* = .087, η^2^﻿ = .055, nor was there a distractor condition × trial type interaction, *F*(1, 52) = 1.23, *p* = .266, η^2^﻿ = .024.

### Experiment 2 discussion

Experiment 1 showed that recognition memory was impaired below baseline only in the one-distractor condition, despite the fact that participants were *faster* to visually locate the target and spent *more* time looking at the target relative to the three-distractor condition. We hypothesized that this was because visual search was easy enough that participants were able to find the target while still processing the *meaningful content* of the distractors in the one-distractor condition. We thus expected that Experiment 2 trials containing meaningful content (i.e., conflict and match trials) in the one-distractor condition would elicit (a) longer look durations to any single distractor, (b) slower target verification times, (c) poorer target categorization accuracy, and (d) impaired target recognition memory. The results of Experiment 2 confirmed these predictions, suggesting that the memory impairment in the context of a single distractor was driven by increased interference from *meaningful* distractors during target encoding. Interestingly, we only observed a memory impairment for conflict trials in the one- relative to the three-distractor condition, suggesting that processing *conflicting* distractors may have interfered with target encoding to a greater degree than processing those that shared category information.

Additionally, based on past studies of perceptual load, we expected that target verification would be slowed by the presence of a conflicting distractor in the one-distractor condition, but we observed no differences in verification times across conflict and match trials. It is possible that distinguishing the target from a distractor that shared category features was equally or even more difficult as distinguishing it from a conflicting distractor (Duncan & Humphreys, 1989), but these overlapping features may not have interfered with target encoding as strongly as conflicting category information. When the distractor category matched the target category or contained no meaningful information, memory performance was similar across the one distractor and the three-distractor conditions. Experiment 2 demonstrates that increasing the amount of distraction from a single distractor to multiple distractors reduces *conceptual* interference during target processing, leading to both more accurate categorization of the target image and *better memory* for the target.

Finally, it is worth noting that our omnibus test of the distractor condition × trial type interaction effect on memory was significant for the proportion of old images remembered with high confidence but not for memory sensitivity (*d*_*a*_). Though the nature of the relationship between memory confidence and memory accuracy is complex and still debated (Roediger, Wixted, & DeSoto, 2012), previous work suggests that in the absence of specific manipulations that affect confidence (i.e., changing the luminance of items between the study and test phase) memory accuracy and memory confidence ratings provided at retrieval lie along a single dimension: the strength of the underlying memory representation (Busey, Tunnicliff, Loftus, & Loftus, 2000). Confidence ratings simply allow for a finer, more sensitive measurement of this underlying strength (Busey et al., 2000). Additionally, given that we had no middle “unsure” rating in our confidence scale, our memory sensitivity measure (*d*_*a*_) was likely diluted by trials in which participants guessed. Examining the proportion of old images remembered with high confidence eliminates these trials (Fukuda & Woodman, 2015; Prince, Daselaar, & Cabeza, 2005), which may have been particularly important in Experiment 2 in which participants were tasked with retrieving more images than in Experiment 1, and in which memory sensitivity was lower overall across conditions.

## General discussion

The present study examined the effects of distractor number and content on visual search and memory for target information. Though participants were slower to find the target and spent less time looking at it as the number of distractors increased, their ability to encode its identity for subsequent recognition memory was impaired to a greater degree when a single distractor was present. Importantly, participants’ memories were impaired to the greatest extent when a single distractor with *conflicting content* was present, indicating that the number of distractors present during visual search interacted with their content to influence encoding and subsequent recognition memory for target images. Under certain conditions, increasing the number of distractors from one to three was better for learning and memory.

It is possible that increasing the number of distractors distributed across multiple spatial locations increased the engagement of selective attention processes during visual search, leading to the reduced strength of any single distractor signal. Previous work has found that engaging selective attention mechanisms involving distractor suppression at encoding results in enhanced encoding of target information (Hutchinson, Pak, & Turk-Browne, 2015; Markant et al., 2015). In the present study, we may have engaged similar selective attention mechanisms by increasing the number of distractors, which would require suppression of multiple distractors and inhibiting return to multiple locations (Snyder & Kingstone, 2000). Actively suppressing multiple distractors spread over more of the visual field may have resulted in increased selective attention engagement and relative reductions in the strength of the distractor signals when more distractors were present (Carrasco, 2011; Gazzaley et al., 2005; Moran & Desimone, 1985; Slotnick, Schwarzbach, & Yantis, 2003; Zhang et al., 2011), leading to more robust target representations in long-term memory (Markant et al., 2015).

However, the results of Experiment 2, in which distractor number and content interacted to influence memory, suggest that increasing the amount of the visual field that was suppressed in and of itself did not lead to better memory performance in the three versus the one-distractor condition. Instead, these data suggest that the more effortful search process elicited by increasing distractor numbers led to stronger suppression of *meaningful information* during target encoding. This explanation is in line with past studies of perceptual load, in which participants demonstrated increased search times for arrays with more distractors, but no differences in search times based on whether the distractor was from the same or different category as the target (Lavie & Cox, 1997; Lavie, 1995). Increasing distractor number can thus result in less interference from distractor content for more difficult search arrays.

Our results are also consistent with previous research suggesting that perceptual load findings may be mediated by selective attention mechanisms involving distractor suppression and target enhancement. Selective attention biases sensory processing to resolve competition between multiple concurrent stimuli (e.g., targets and distractors) for representation in sensory cortex (Carrasco, 2011; Desimone & Duncan, 1995). Torralbo and Beck (2008) posited that as the number of distractors in an array increases, a larger attentional bias is needed to resolve competition in favor of the target stimulus. “Load” may simply index the relative need for selective attention to resolve competition between stimuli, with longer search times in “higher load” conditions reflecting greater competition between targets and distractors (Torralbo & Beck, 2008). When a stronger bias is needed to detect the target within large or complex search arrays, individuals more strongly engage selective attention mechanisms to suppress distractor signals (Parks, Beck, & Kramer, 2013; Torralbo & Beck, 2008). In the present experiments, the increased load of the three-distractor condition may have similarly elicited greater competition and required stronger suppression of the distractors for effective target detection.

Increasing the number of distractors while keeping the trial time consistent across conditions may have also resulted in more competition for attention between the distractors themselves (Scalf, Torralbo, Tapia, & Beck, 2013; Tsal & Benoni, 2010), causing participants to spend less time processing any single distractor. In the present study participants spent equal amounts of time looking at all of the possible distractor locations the one and three-distractor conditions, but spent more time looking at any single distractor in the one-distractor condition. In the three-distractor condition, they divided their attention across multiple distractor locations. This explanation is also consistent with the idea that increased *spatial* or *perceptual* competition between items in a search array may result in reduced distractor signal strength and less interference from the conceptual content of any distractor during target processing.

Critically, the attentional bias required to resolve target/distractor competition that has been identified in previous perceptual load studies is the same mechanism implicated in previous work showing effects of selective attention engagement on memory encoding. The present study bridges these two bodies of work by showing that increasing the number of distractors present in a learning environment both reduces interference from meaningful distraction during target processing, as seen in previous perceptual load studies, *and* reduces the extent to which competing, meaningful distraction impairs target recognition memory. Our data indicate that the amount and type of distraction present in a learning environment interact to influence the degree to which target information can be learned and remembered, potentially by modulating the degree to which selective attention processes can resolve interference from competing distractors.

The memory impairment we observed in the context of a single distractor was specifically driven by poorer memory for targets encoded in the presence of conflicting distractors. This result differs from those of three recent studies that observed *enhanced* memory for targets encoded in conflict conditions. In these studies, targets and distractors were overlaid or interleaved, and memory for targets was enhanced when the distractor category conflicted with that of the target and the required response (Krebs et al., 2013; Rosner, D’Angelo, MacLellan, & Milliken, 2015; Rosner & Milliken, 2015). The researchers interpret these data as evidence that increasing the demands on selective attention at encoding results in a stronger attentional bias toward relevant target features and away from competing distraction. Importantly, this interpretation aligns with our hypothesis that we observe memory impairments in the context of a single distractor because the easier search process elicited by the presence of only a single distractor does not engage target enhancement and distractor suppression mechanisms to the same extent as the more difficult search process in the three-distractor case. One possible explanation for the discrepancy between our results and these previous findings may have to do with the fact that the targets and distractors in our study were spatially distributed as opposed to overlapping, resulting in different interactions between spatial and feature-based attention on recognition memory (Hayden & Gallant, 2009). Future work should further explore the contexts in which conflict benefits and hurts memory.

The present data show that people experience greater interference from conceptual distraction when *a single* distractor is present, and critically, that this interference impairs learning and memory. This suggests that if distraction is present, increasing numbers of distractors can benefit memory by reducing conceptual interference during target encoding. Detecting and categorizing a target in an environment flooded with visual noise may be more difficult than categorizing a target presented alongside only one distractor, but the processes we engage when we attempt to perceive and identify visual stimuli in cluttered environments may be beneficial for learning.

## Footnotes

1 When analyzing our data, we discovered that for the first 24 participants tested, two images were inadvertently presented twice in different conditions across the experiment. Trials containing these images as either targets or distractors (between 0 and 4 trials per participant) were excluded from all analyses.
